# Supplementary material for: Versatile stochastic dot product circuits based on nonvolatile memories for high performance neurocomputing and neurooptimization
Source: Nat Commun. 2019 Nov 8;10:5113. doi: 10.1038/s41467-019-13103-7 (PMC6841978; doi:10.1038/s41467-019-13103-7)
Supplement: Supplementary file 1 — Supplementary Information [file 41467_2019_13103_MOESM1_ESM.pdf]

Supplementary Information for

Versatile Stochastic Dot Product Circuits Based on Nonvolatile Memories for High Performance Neurocomputing and Neurooptimization

Mahmoodi et al.

Supplementary Figure 1

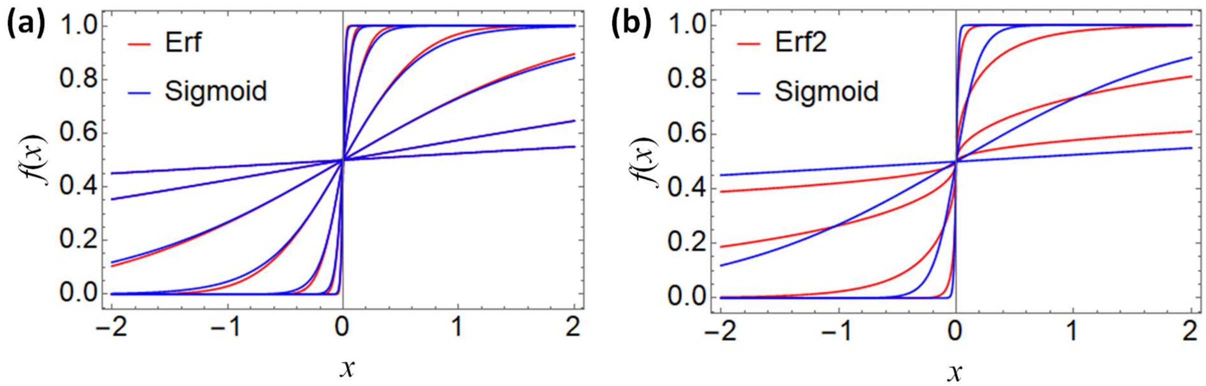

Supplementary Figure 1. Sigmoid function approximation. Left hand side and right hand side of (a) Supplementary Equation 1 shown as a function of an argument x/T with $T = 10$, 3.33, 1, 0.33, 0.1, 0.033, 0.01, and (b) Supplementary Equation 2 shown for $T = 10$, 1, 0.1, 0.01.

Supplementary Figure 2

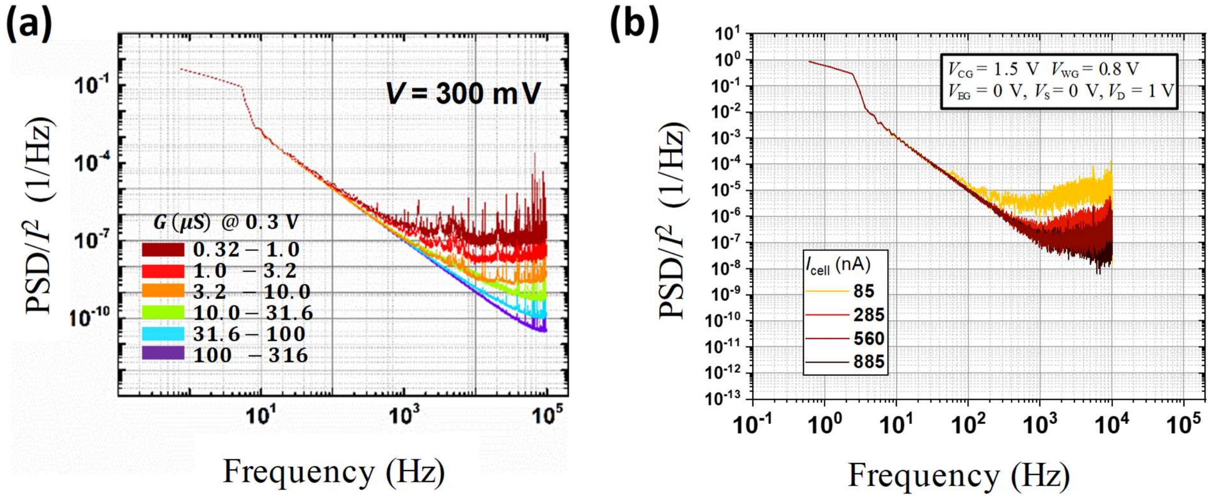

Supplementary Figure 2. Noise characterization. Normalized current power spectral density for (a) memristor and (b) floating gate memory experimental setups. The spectra were obtained over range of currents of interest by changing the memory device conductances. For memristors, the spectra were collected for 324 devices in the crossbar, binned in 6 conductance ranges and averaged, while panel b shows representative spectrum, measured on a specific floating gate device. In both cases, the flat part of the spectrum is due to the read-out circuitry of the experimental setup, and hence such noise can be considered as an external noise in the equivalent circuit in Figure 1a. In particular, the noise is mostly generated at the custom-made switching matrix in floating gate memory setup (Supplementary Fig. 6), while it is contributed by the combination of custom PCB, switching matrix, and B1530 tool in memristor experiments (Supplementary Fig. 5). The corresponding root mean square of the time series of the measured current is roughly 300 nA for memristor setup and 1.5 μA for flash memory setup at the studied bandwidths – see also insets in Figures 2c and 3b. (Note that the SNR_{max} data are smoother in inset of Figure 3b, because of the single-ended sensing in flash memory experiments.)

Supplementary Figure 3

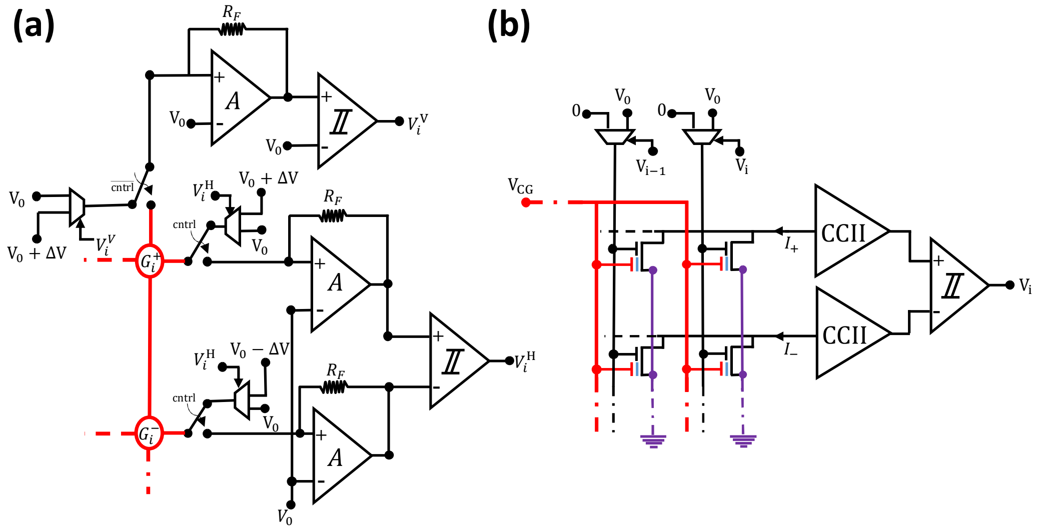

Supplementary Figure 3. Possible implementation of peripheral circuits. (a) Bidirectional voltage-mode neuron design for the memristor RBM network and (b) the current-mode implementation of neurooptimization with embedded NOR flash memory. In panel a, outputs (in both direction) are $f(\sum_{i=1}^{N} V_i(G_i^+ - G_i^-))$ where V_i is either V_0 or $V_0 \pm \Delta V$ depending on the state of the ith neuron and the neuron type (visible or hidden), while *f*() is a step function activation function realized by a high gain comparator. In panel b, a current conveyor is used for current sensing and a current-mode comparator is utilized to perform the binary activation (see e.g., Ref. [16] for a transistor-level implementation of both circuits). For clarity, both panels do not include registers (which would be needed for discrete time / state networks), and tuning circuitry.

Supplementary Figure 4

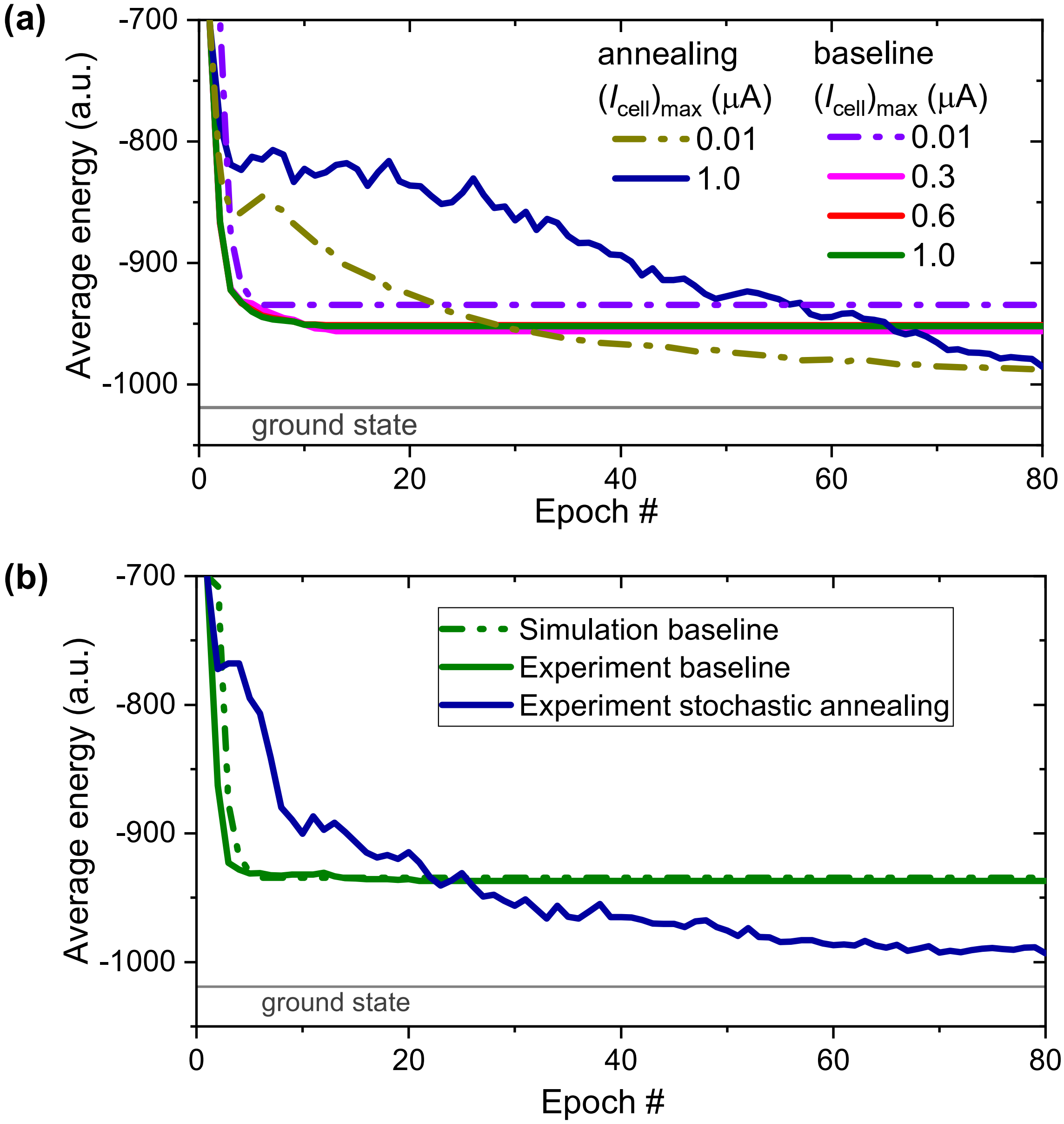

Supplementary Figure 4. Additional data for the studied neurooptimization problem. (a) Experimental (solid lines) and SPICE simulation (dot-dash) results for flash memory circuits. Inset shows maximum value of currents used in mapping weights to the synaptic conductances. Simulations are performed assuming intrinsic noise of field effect transistor at 55 nm process at 100 MHz bandwidth operation for the stochastic approach, and noise-free operation for the baseline approach. (b) Experimental (solid lines) and simulation (dot-dashed) results for memristor-based circuits. The experiments were performed on 6×8 subarray of the same crossbar circuit, which was used for experiment shown in Figure 2, with 1 MHz sampling bandwidth. The neurooptimization network weights were mapped to [10 μS, 50 μS] range of memristors' conductances, which were programmed with <5% tuning error. The shown experimental data are averaged over 160 runs (10 runs for each of the 16 initial states). Note that the data for $(I_{cell})_{max}$ = 1 μA experiment in panel a and the simulation results in panel b are the same as in Fig. 3e and are shown for comparison.

Supplementary Figure 5

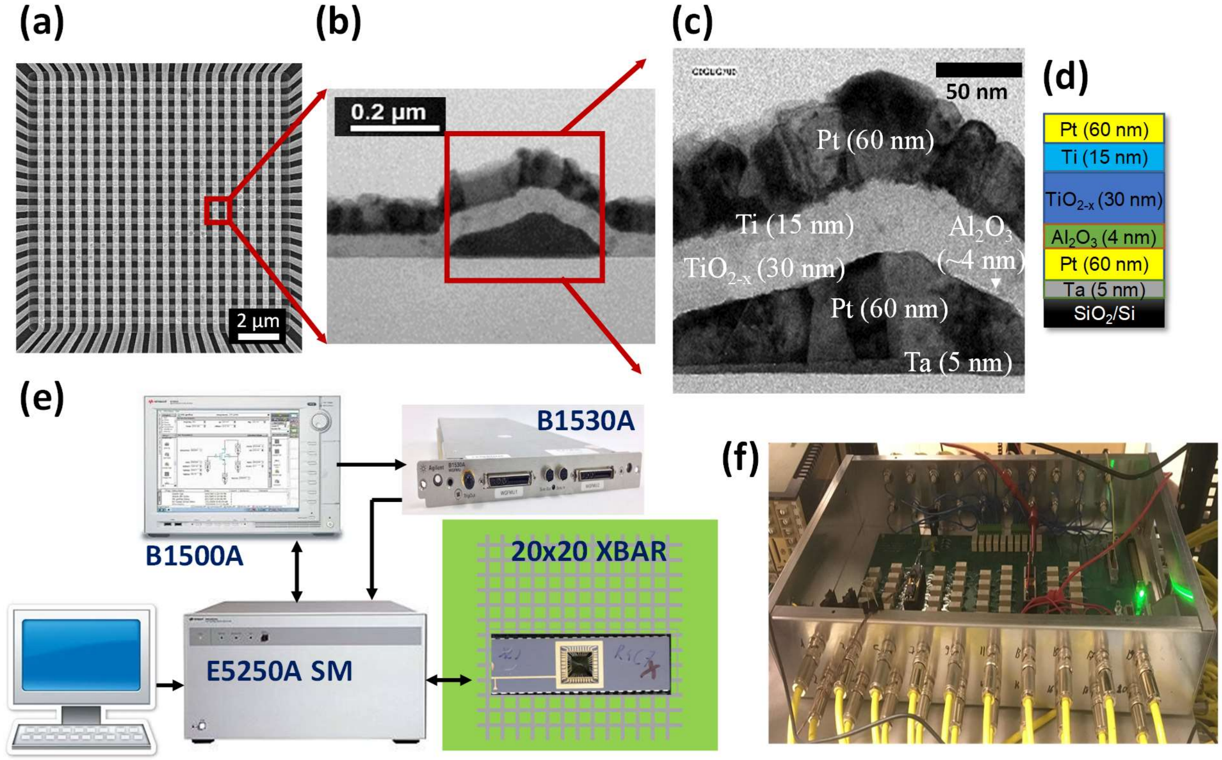

Supplementary Figure 5. Experimental setup for memristor circuits. (a) Top-view SEM image, (b, c) cross-section TEM images, and (d) the device stack of the 20×20 passively integrated TiO_2 memristor crossbar used in the restricted Boltzmann machine demo. (e) The experimental setup comprising of a personal computer to control parameter analyzer B1500A, arbitrary waveform generator B1530A, and low-leakage Agilent E5250A switch matrix. (f) Memristor chip mounted on custom printed circuit board.

Supplementary Figure 6

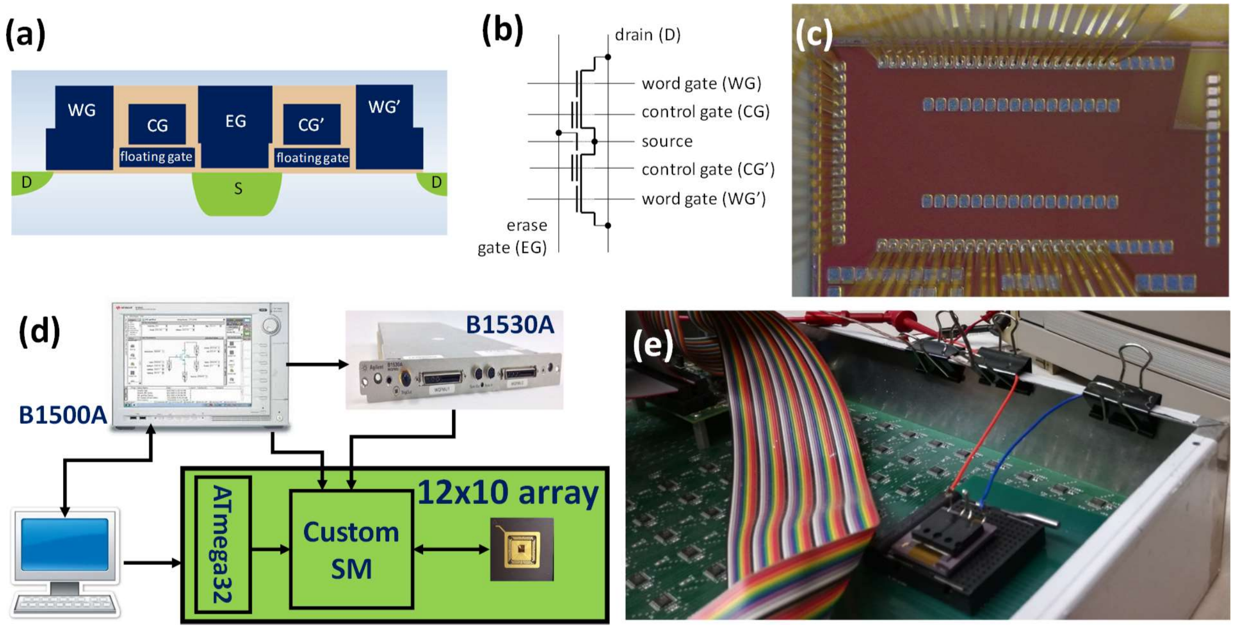

Supplementary Figure 6. Experimental setup for floating-gate memory circuits. (a) Cross-section of SST's 55 nm ESF3 NOR flash supercell incorporating two floating-gate transistors with a common source and erase gates. (b) The schematics of supercell. (c) The micrograph of a 12×10 modified NOR flash memory array fabricated in GF's 55 nm LPE CMOS process. (d) The characterization setup used in this work for the demonstration of neurooptimization experiments. The setup includes a personal computer to control the parameter analyzer B1500A, waveform generator B1530A, and a custom-made printed circuit board, which hosts a microcontroller to control the state of the custom switch matrix (a bank of ADG1438 analog switches). (e) The chip mounted on the printed circuit board.

Supplementary Figure 7

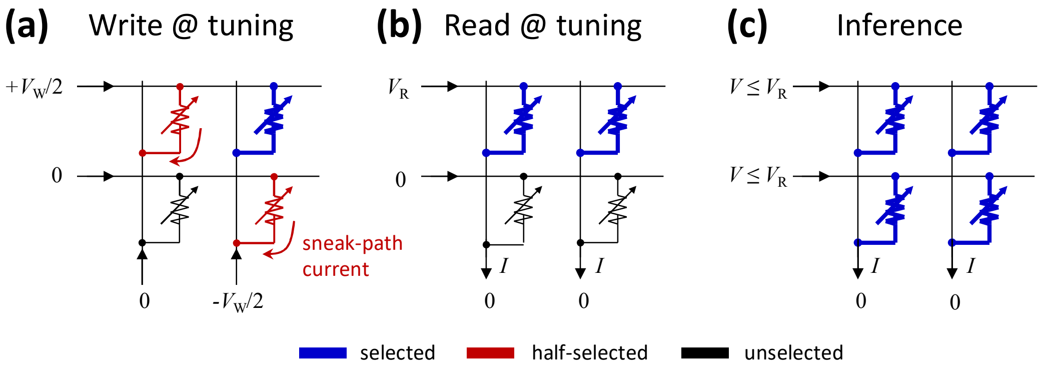

Supplementary Figure 7. Crossbar circuit biasing and sneak path currents. Sneak-path currents for the three distinctive operation in the ex-situ-trained neuromorphic circuits: (a) writing with "V/2-biasing" scheme and (b) reading the state at the conductance tuning step, and (c) inference operation, shown for simplicity for 2×2 fragment of the crossbar circuit. Smaller sneak-path currents can also flow via unselected devices with significant voltage drops across crossbar lines.

Supplementary Table 1. Comparison of neurooptimization hardware.

Device	Main result / type of demo	Ref	Comments
Magnetic	Experimentally measured ground states for the network consisting of up to 3 coupled magnetic devices with fixed coupling	[2]	Suitable for implementation of neurons but not for much more numerous synapses. Solutions for efficient, scalable dot-product circuits based on magnetic devices have yet to be demonstrated.
	Simulations of graph coloring and maximum cut problem	[3]	
	Simulations of a network solving 16-city traveling salesman problem, based on experimentally verified stochastic binary neuron	[4]	
	Experimental demo of two-node directed probabilistic network based on discrete stochastic binary neurons	[5]	
CMOS	Experimental results for solving maximum-cut problem with 20K-spin Ising network based on fully-integrated 65-nm 12-mm^2 260k SRAM-cell chip	[6]	The most mature technology with the most advanced demonstrations. The main disadvantage is low integration density.
	Experimental results for solving maximum-cut problem with 2×30K-spin Ising network based on two PCB-connected fully-integrated 40-nm 23.65-mm^2 SRAM-based chips	[7]	
	Experimental results for solving 3-SAT problem with 50 variables and 218 clauses and solving optimal coloring of 5×5 queen graph based on 180-nm chip with 64×32 array of spiking LIF neurons	[8]	
Josephson junction	Experimentally measured evolution of 1D spin system based on 8 superconducting flux qubits (with evidence of quantum annealing)	[9]	The most perspective technology due to quantum speedup though prospects for scaling to the larger systems are unclear
	Experimentally measured ground state of random spin glass problems based on 108-qubit D-Wave One system (with evidence of quantum annealing)	[10]	
Photonics	Experimental results for solving various problems with 100 spin / 10,000 spin-spin connections Ising machine based on degenerate optical parametric oscillators	[11]	Contemporary implementations are slow due to high overhead of the electronic feedback used for updating spatial light modulator
	Experimental results for 1D 10,000-spin Ising network based on degenerate optical parametric oscillators	[12]	
	Experimental results for solving max-cut problems with up to 2,000 nodes with Ising network based on degenerate optical parametric oscillators	[13]	
	Experimentally measured ground states for various spin systems based on a network with up to 45 locally coupled polaritons	[14]	
Memristor and eFlash	Simulations of memristor-based neural network for solving TSP problem, including chaotic annealing, based on experimental data from discrete NbO_2 devices	[15]	Most promising implementations are based on hybrid CMOS / memristor circuits. eFlash approach is more mature but also less dense compared to memristor ones.
	Experimentally measured evolution of the network state for 10-input/8-hidden neuron RBM and experimental results for solving 4-node graph partitioning problem based on integrated 55-nm 12×10-cell floating gate transistor array and ~250-nm 20×20 passive metal-oxide memristor crossbar circuits, with hw-injected noise and emulated periphery of crossbar circuits.	This work	

Supplementary Table 2. Biasing conditions. Biasing details for the considered neurooptimization approaches.

	V_D	V_S	V_{EG}	V_{CG}		V_{WG}	
	-	-	-	fixed weights	variable weights	fixed weights	variable weights
**Baseline**	1	0	0	1.5 V	-	0.8	--
**Adjustable**	1	0	0	1.5 V	1.5 V	1.2	1.2 → 0.2 (adjustable node), 1.2 → 1 (bias)
**Chaotic**	1	0	0	1.5 V	1.5 V	0.8	1.2 → 0
**Stochastic**	1	0	0	-	1 V → 2 V	0.8	0.8

<u>Supplementary Note 1</u>: Approximations for sigmoid probability distribution function of stochastic neuron

Equations 1 and 3 of the main text are related via the following approximation

$$\frac{1}{1+\exp(-x)} \approx \frac{1}{2} + \frac{1}{2}\mathrm{erf}\left(\frac{\sqrt{\pi}x}{4}\right), \tag{1}$$

which is very accurate across wide range of argument x (Supplementary Fig. 1a). In fact, the relative error is always smaller than 2%.

On the other hand, approximation

$$\frac{1}{1+\exp(-x)} \approx \frac{1}{2} + \frac{1}{2}\mathrm{erf}\left(0.187\sqrt{x}\right) \tag{2}$$

is useful for the shot noise regime. Assuming noise variance $\sigma^2 = \alpha I$, the adjusted effective temperature $T' = 0.07\alpha/I_{\max} \equiv 0.11\alpha/\sigma_{\max}\times T$, where $T = \sqrt{2\pi}\sigma_{\max}/(4I_{\max})$ is defined similarly to Eq. 3 of the main text, can be used to ensure less than 10% relative error over all values of x between the two functions (Supplementary Fig. 1b). Note that approximation with such adjusted temperature overestimates probability density function at currents close to $I_{\max}$ (hence actual effective temperature is slightly cooler), while underestimated it (i.e., actual effective temperature is hotter) at currents close to 0 (Supplementary Fig. 1b).

The intermediate cases, with mixture of shot and thermal noises can be similarly approximated with better than 10% relative accuracy.

<u>Supplementary Note 2</u>: Solving graph partitioning problem with Hopfield network

Let us consider a graph (U, E) with N nodes, node weights w_i, and edge weights e_{ij}. Since each node will be uniquely mapped to the corresponding neuron, U_i is also used to define the state of i-th neuron. The problem is to partition the graph into two partitions of nearly equal weight such that the cutsize, the number of edges with an end point in each partition, is minimized.

To solve this problem, let us consider discrete-time discrete-state recurrent neural network. The intuitive energy function is given by

$$E = \alpha \sum_{i=1}^{n}\sum_{j=1}^{n} e_{ij}(U_i + U_j - 2U_iU_j) + \sum_{i=1}^{n}\sum_{j=1}^{n} w_iw_j(1 - U_i - U_j + 2U_iU_j), \tag{3}$$

where the first term minimizes the weighted sum of edges which belong to the cut, and the second term will have a minimum value when the sum of node weights assigned to the two partitions are equal, while $\alpha = 0.5$ is a constant representing relative importance of these two terms [1]. By dropping constant terms and rearranging this expression, a more convenient energy function for neural network, for which diagonal weights should be zero to ensure that energy is decreasing during state updates, is

$$E = -\frac{1}{2}\sum_{i=1}^{n}\sum_{j=1,j\neq i}^{n}\left(2e_{ij} - 4w_iw_j\right)U_iU_j - \sum_{i=1}^{n} U_i\left(2w_i\sum_{j=1}^{n} w_j - 2{w_i}^2 - \sum_{j=1}^{n} e_{ij}\right). \tag{4}$$

(Note that there is a mistake in Ref. [1] in that $-2w_i^2$ term in bias weights is missing.) This equation directly defines neural network array and bias weights:

$$T_{ij} = 2e_{ij} - 4w_i w_j, \quad T_i^{\text{b}} = 2w_i \sum_{j=1}^{n} w_j - 2w_i^2 - \sum_{j=1}^{n} e_{ij}, \tag{5}$$

The corresponding utilized synaptic weights in the floating-gate implementation are

$$I_{\text{cell}} = \begin{pmatrix} 0 & -140 & -478 & -500 \\ -140 & 0 & -508 & -552 \\ -478 & -508 & 0 & -622 \\ -500 & -552 & -622 & 0 \end{pmatrix} \times \frac{(I_{\text{cell}})_{\max}}{622}, \quad I_{\text{cell}}^{\text{b}} = \begin{pmatrix} 559 \\ 600 \\ 804 \\ 837 \end{pmatrix} \times \frac{(I_{\text{cell}})_{\max}}{837} \tag{6}$$

where $(I_{\text{cell}})_{\max}$ is the largest cell current, which, as discussed in main text, is controlled by adjusting WG and/or CG line voltages. Note that bias and array weights are always of different signs for the graph partitioning problem and to increase dynamic range and improve nonlinearity, these two group of weights are normalized differently. This can be readily implemented in a hardware by having different gains for the positive and negative pre-amplifiers in differential sensing circuitry.

Supplementary Note 3: Towards higher density and capacity passive crossbar circuits

As with the deterministic mixed-signal inference accelerators – see, e.g. Refs. 16, 19, performance of the proposed hardware could be improved by utilizing larger and denser crossbar circuits. The main challenge for that is resistive (IR) drop across crossbar lines, which can be loosely defined as non-negligible voltage drop across crossbar lines, leading to smaller voltages applied across crosspoint devices. Non-negligible sneak-path currents via half-selected devices (and to lesser degree via unselected devices) result in larger IR drops for the write operation. However, by design, sneak-path currents never occur at inference operation (Supplementary Fig. 7).

To estimate the impact of IR drops in write phase of the tuning algorithm, let us assume 'V/2-biasing' scheme (Supplementary Fig. 7). The largest current via electrodes of $N \times N$ crossbar circuit, without taking into account IR drop, can be roughly estimated as

$$(I_{\text{line}}^{\text{write}})_{\max} \approx (N\text{-}1)V_{\text{W}}/2\ G_{\text{on}}(V_{\text{W}}/2) + I(V_{\text{W}}) \approx N\ V_{\text{W}}/2\ G_{\text{on}}(V_{\text{W}}/2), \tag{7}$$

where V_{W} is a write (set or reset) voltage, G_{on} is the largest memory cell conductance used at inference operation. The first and the second terms in Supplementary Equation 7 are due to sneak-path currents via half-selected devices and the current via selected device at resistive switching, respectively, while the approximation is valid for larger N and non-negligible sneak-path currents. The similarly estimated largest crossbar line current at inference operation, performed at non-disturbing voltages $|V| \leq V_{\text{R}}$, is

$$(I_{\text{line}}^{\text{infer}})_{\max} \approx N\ V_{\text{R}}\ G_{\text{on}}(V_{\text{R}}). \tag{8}$$

(The worst case line current at read operation is similar to inference. However, read operation is less challenging because of smaller output currents and the possibility for taking into account IR drops into the measurement current values.)

The line currents can be used to estimate the worst case normalized difference between the voltage applied at the periphery of the crossbar and the voltage dropped across the crosspoint device as $(\Delta V/V)_{\max} \approx 2(I_{\text{line}})_{\max} N/(VG_{\text{wire}})$, where G_{wire} is a conductance of full-pitch-long crossbar

line segment and factor of 2 is due to the IR drops at both lines leading to the crosspoint devices. Hence, the normalized errors in the applied voltage for write and inference operations are, respectively,

$$(\Delta V_W/V_W)_{max} \approx 2N^2 G_{on}(V_W/2)/(2G_{wire}), \quad (9a)$$

$$(\Delta V_R/V_R)_{max} \approx 2N^2 G_{on}(V_R)/G_{wire}. \quad (9b)$$

As it is evident from Supplementary Equation 9, the IR drop increases quadratically with N, and the general solution to this problem is to increase line conductance and/or decrease conductance ranges in the crosspoint devices.

Because of the crude lumped model analysis, Supplementary Equation 9 overestimates the voltage errors. In our earlier work (Supplementary Note 1 of Ref. 20), we provided accurate quantitate estimates for the acceptable ratio of device to wire conductances, which would be required for the correct operation assuming the same technology crossbar circuits. Let us here instead compare the impact of IR drop on the write and inference operations. The vector-by-matrix multiplication error at the inference operation due to IR drop, in the absence of other circuit and device non-idealities, is proportional to $(\Delta V_R/V_R)_{max}$. Performing computation with the effective p-bit precision requires $(\Delta V_R/V_R)_{max} \leq 1/2^{p+1}$, which is 3% for $p = 4$. (Note that because IR drop is input-dependent, the error cannot be practically compensated by adjusting crosspoint conductances.) On the other hand, much worse IR drops can be tolerated at write operation due to the feedback in the write-verify tuning algorithm, which does not require applying precise voltage pulses across crosspoint devices. If needed, larger voltages would be applied to the lines during tuning to compensate for IR drop, and the amplitude of write voltages is bounded only by the requirement of not disturbing already tuned half-selected devices [17]. Assuming "V/2-biasing" scheme and 20% standard deviation of switching voltage thresholds, which is representative of the considered crossbar circuits (Fig. 2 of Ref. 20), results in $100\times(\Delta V_W/V_W)_{max} \leq \sim 30\%$. Analyzing the Supplementary Equations 9a and 9b using 30% and 3% allowable voltage errors at write and inference operations, and typical $V_R = 0.1$ V, $V_W = 1.2$ V, and $G_{on}(0.6\text{V})/G_{on}(0.1\text{V}) \leq 2$ for the utilized memristors, it is clear that IR drop problem is the most severe at inference operation, even for suboptimal "V/2-biasing" scheme. Therefore, reducing device conductances and/or increasing wire conductance to the acceptable values to ensure correct implementation of inference operation would automatically result in acceptable IR drops for write operation at tuning step.

Finally, let us note that there are enormous reserves for decreasing device to wire conductance ratio for the developed memristor technology. For example, we have recently developed similar-density crossbar circuits based on the same TiO_2/Al_2O_3 resistive switching stack but with ~10× higher G_{wire} by utilizing CMOS-foundry-compatible etch-back process [18]. We also expect that $G_{on}(V_R)$ for the utilized devices would scale inversely proportional to the device cross-section area – see Supplementary Note 1 from Ref. 20 for more details.

Supplementary References

[1] Ramanujam, J. & Sadayappan, P. Mapping combinatorial optimization problems onto neural networks. *Inf. Sci.* **82**, 239-255 (1995).

[2] Debashis, P. et al. Experimental demonstration of nanomagnet networks as hardware for Ising computing. in *IEEE International Electron Devices Meeting (IEDM)* 3.4.1–3.4.4 (IEEE, 2016).

[3] Shim, Y., Jaiswal, A. & Roy, K. Ising computation based combinatorial optimization using spin-Hall effect (SHE) induced stochastic magnetization reversal. *J. Appl. Phys.* **121**, 193902 (2017).

[4] Sutton, B., Camsari, K.Y., Behin-Aein, B. & Datta, S. Intrinsic optimization using stochastic nanomagnets. *Sci. Rep.* **7**, 44370 (2017).

[5] Ostwal, V., Debashis, P., Faria, R., Chen, Z. & Appenzeller, J. Spin-torque devices with hard axis initialization as stochastic binary neurons. *Sci. Rep.* **8**, 16689 (2018).

[6] Yamaoka, M. et al. A 20k-spin Ising chip to solve combinatorial optimization problems with CMOS annealing. *IEEE J. Solid-State Circuits* **51**, 303-309 (2015).

[7] Takemoto, T. et al. 2.6 A 2× 30k-spin multichip scalable annealing processor based on a processing-in-memory approach for solving large-scale combinatorial optimization problems. in *IEEE International Solid-State Circuits Conference (ISSCC)* 52-54 (IEEE, 2019).

[8] Mostafa, H., Muller, L.K. & Indiveri, G. An event-based architecture for solving constraint satisfaction problems. *Nat. Commun.* **6**, 8941 (2015).

[9] Johnson, M.W. et al. Quantum annealing with manufactured spins. *Nature* **473**, 194 (2011).

[10] Boixo, S. et al. Evidence for quantum annealing with more than one hundred qubits. *Nat. Phys.* **10**, 218 (2014).

[11] McMahon, P. et al. A fully programmable 100-spin coherent Ising machine with all-to-all connections. *Science* **354**, 614-617 (2016).

[12] Inagaki, T. et al. Large-scale Ising spin network based on degenerate optical parametric oscillators. *Nat. Photonics* **10**, 415 (2016).

[13] Inagaki, T. et al. A coherent Ising machine for 2000-node optimization problems. *Science* **354**, 603-606 (2016).

[14] Berloff, N. et al. Realizing the classical XY Hamiltonian in polariton simulators. *Nat. Mater.* **16**, 1120 (2017).

[15] Kumar, S., Strachan, J.P. & Williams, R.S. Chaotic dynamics in nanoscale NbO_2 Mott memristors for analogue computing. *Nature* **548**, 318 (2017).

[16] Mahmoodi, M.R. & Strukov, D.B. An ultra-low energy internally analog, externally digital vector-matrix multiplier circuit based on NOR flash memory technology. in *ACM Design Automation Conference (DAC)* 22 (ACM, 2018).

[17] Strukov, D.B. Tightening grip. *Nat. Mater.* **17**, 293-295 (2018).

[18] Kim, H., Nili, H., Mahmoodi, M. & Strukov, D. 4K-memristor analog-grade passive crossbar circuit. Preprint at https://arxiv.org/abs/1906.12045 (2019).

[19] Bavandpour, M. et al. Mixed-signal neuromorphic inference accelerators: Recent results and future prospects. in *IEEE International Electron Devices Meeting (IEDM)* 20.4.1-20.4.4 (IEEE, 2018).

[20] Merrikh Bayat, F. et al. Implementation of multilayer perceptron network with highly uniform passive memristive crossbar circuits. *Nat. Commun.* **9**, 2331 (2018).
